# Identification and Expression Pattern Analysis of Cuticular Protein Gene Family in *Monochamus alternatus* (Coleoptera: Cerambycidae)

**DOI:** 10.3390/biology15141174

**Published:** 2026-07-16

**Authors:** Xiaoman Ning, Amin Zhu, Jinrui Zhu, Bin Liu

**Affiliations:** 1 Guangxi Colleges and Universities Key Laboratory for Cultivation and Utilization of Subtropical Forest Plantation, School of Forestry, Guangxi University, Nanning 530004, China; mnwlkq2020@163.com (X.N.); 16655181621@163.com (A.Z.); z18365115120@163.com (J.Z.); 2 Guangxi Youyiguan Forest Ecosystem National Observation and Research Station, Pingxiang 532600, China

**Keywords:** bioinformatics analysis, expression analysis, phylogenetic tree analysis, developmental-stage-specific expression, conserved motif

## Abstract

The pine sawyer beetle (*Monochamus alternatus* Hope, 1842) is a major vector of the pinewood nematode [*Bursaphelenchus xylophilus* (Steiner & Buhrer, 1934) Nickle, 1970]. A total of 119 cuticular protein (CP) genes were identified from the *M. alternatus* genome, belonging to nine families, namely 34 CPR-RR1, 39 CPR-RR2, 6 CPAP1, 7 CPAP3, 2 CPF, 3 CPFL, 4 CPCFC, 14 CPU, and 10 TWEEDLE, with the CPR family being the most abundant (73 genes, 61.34%). Comparative genomic analysis across six insect orders revealed that the CP gene count in *M. alternatus* (119) falls within the range observed among other Coleoptera species (119–173). Synteny analysis revealed seven tandem duplication events on chromosome 6 within the *M. alternatus* genome and 16 conserved syntenic gene pairs between *M. alternatus* and *Tribolium castaneum* (Herbst, 1797), distributed across six chromosomes. Expression profiling across larval, pupal, and adult stages revealed that CP genes within the same family exhibited distinct developmental-stage-specific expression patterns. Among the CPR-RR1 family, four genes (Malt029850.1, Malt047022.1, Malt029928.1, and Malt029871.1) showed higher expression levels; among the CPR-RR2 family, three genes (Malt014886.1, Malt031777.1, and Malt014882.1) showed higher expression. Quantitative real-time PCR validation confirmed that two CPR-RR1 genes (Malt029850.1 and Malt047022.1) were highly expressed in larvae, two CPR-RR1 genes (Malt029928.1 and Malt029871.1) were highly expressed in adults, and three CPR-RR2 genes (Malt014886.1, Malt031777.1, and Malt014882.1) were expressed predominantly in pupae.

## Simple Summary

The pine sawyer beetle is a major pest that damages pine trees and spreads pine wilt disease, a serious illness that kills pine trees around the world. Like most insects, this beetle has a hard outer body covering that contains special proteins essential for its growth and survival. This study aimed to identify all the genes responsible for producing these proteins in the beetle and to investigate how they behave during different life stages. By combining computational analysis with laboratory experiments, the research team identified a large set of genes encoding these proteins, which were classified into several distinct families. They also examined how active these genes were in young beetles, pupae, and adult beetles. The results revealed that different genes exhibited increased activity at different life stages. Understanding these patterns helps scientists learn how the beetle develops, which could contribute to the development of new environmentally friendly strategies to protect forests from this destructive pest without relying heavily on chemical sprays.

## 1. Introduction

The insect cuticle is the first line of defense against the external environment, and insects develop pesticide resistance by regulating cuticle permeability [1]. The main components of the insect cuticle are cuticular proteins (CPs) and chitin. Among them, CPs are key structural components in insects, and their types and quantities vary between species or at different developmental stages of the same species [2,3]. In 1982, Snyder et al. first reported five CP genes (DmelCP1-5) in *Drosophila melanogaster* Meigen, 1830 [4]. Subsequently, researchers identified a total of 228 CP genes from the *D. melanogaster* genome [5]. With the completion of genome sequencing for insects such as the silkworm *Bombyx mori* (Linnaeus, 1758) [6,7,8], *Aedes aegypti* (Linnaeus, 1762) [9,10], *Anopheles gambiae* Giles, 1902 [11,12,13], *Apis mellifera* Linnaeus, 1758 [14], and *Tribolium castaneum* [15,16,17,18], a large number of CP genes have been identified, providing important resources for the study of the evolution and function of the CP gene family, although their numbers vary considerably among different species.

Currently, the NCBI (National Center for Biotechnology Information) database contains thousands of insect CP sequences with detailed descriptions [19,20,21]. Numerous studies have confirmed that CPs play critical physiological roles in insect growth and development, cuticle sclerotization, locomotion, body shape formation, innate immunity, and pesticide resistance [16,17]. To understand the molecular basis of insecticide penetration resistance and to explore dsRNA-based control strategies against *M. alternatus*, the first step is to annotate cuticle proteins (CPs) more accurately.

Based on conserved domains in amino acid sequences [5,22], insect CPs are classified into 12 families [22]. The largest CP group is a family with the Rebers and Riddiford motif (CPR) that contains a core 28-amino acid sequence within a 63-amino acid consensus sequence (pfam00379) [22,23]. CPRs are further classified into three subtypes: RR-1, RR-2, and RR-3 [24,25,26]. Other CP families with conserved sequence motifs include CPs analogous to peritrophins (CPAP) [17], CPs with a 42- to 44-amino acid motif (CPF) [26,27], CPF-like (CPFL) [26], CPs with a Tweedle motif (TWEEDLE) [20], CPs with two or three repeats of a C-X (5)-C motif (CPCFC) [22], and CPs with an 18-amino acid motif [25]. CPLCA, CPLCG, CPLCW, and CPLCP are low-complexity families with their own distinct sequence features (with alanine, glycine, tryptophan, and proline, respectively) [11]. Additionally, low-complexity proteins containing repeats of AAP (A/V), P (V/Y), GYGL, or GLLG are also found in the cuticle [22].

*Monochamus alternatus* is a significant pest of coniferous trees, primarily damaging *Pinus massoniana* Lamb., 1803; *Pinus tabulaeformis* Carrière, 1867; and *Pinus thunbergii* Parl., 1868, among others [28]. It is also the main vector for pine wilt disease [29,30]. Guan found that deletion of the Tweedle D1 gene affects the body morphology of Drosophila, causing larvae and pupae to shorten [20]. *Leptinotarsa decemlineata* Say, 1824 increases its environmental adaptability by regulating the expression of cuticle protein genes LdecGRP1, LdecGRP2, and LdecGRP3 [31]. Dittmer et al., using proteomics and transcriptomics, identified many CPR-RR2 genes in *T. castaneum*, while CP-RR1 genes are concentrated in the soft hind wings, indicating that CP gene types have an important influence on the epidermal physical properties [15]. In summary, insect CP genes play important roles in growth and development.

Currently, research on cuticular proteins associated with *M. alternatus* remains limited. Exploring how to effectively inhibit cuticle formation—thereby killing the insect through methods such as chemical control—is a research hotspot in the field of insect physiology and biochemistry. Utilizing the whole-genome data of *M. alternatus*, this study aims to identify the cuticular protein (CP) family genes, conduct comparative analysis with CP genes from other insect species, reveal the evolutionary relationships of MaltCP genes, and investigate the expression patterns of the CP gene family across different developmental stages of *M. alternatus.* The research findings will not only lay a foundation for functional studies of MaltCP genes but also provide theoretical guidance for the control of this beetle species.

## 2. Materials and Methods

### 2.1. Insect Source

*Monochamus alternatus* larvae were collected from Xingning District, Nanning City, Guangxi. Weakened pine trees infested by *M. alternatus* in *P. massoniana* forests were cut into short sections and split open with an axe to collect the larvae from the wood. After being brought back to the laboratory, the larvae were reared in an artificial climate incubator set at 25 ± 1 °C, 70 ± 5% humidity, and a photoperiod of 12 h L–12 h D. To obtain different larval instars, newly hatched larvae were individually transferred to plastic containers (diameter 5 cm, height 8 cm) and fed an artificial diet in the incubator. The developmental stages were monitored daily, and the 1st- to 5th-instar larvae (L1–L5) were distinguished based on head capsule width and body size, according to previously described criteria [32]. Larvae were fed an artificial diet in the incubator until they reached the pupal and adult stages. The diet was prepared according to Chen et al., 2017 [33], with the following composition: pine sawdust 100 g, agar 40 g, sucrose 20 g, yeast extract 12.5 g, sodium benzoate 2 g, potassium sorbate 1 g, 0.5 mol/L H_2_SO_4_ 10 mL, wheat germ powder 25 g, cholesterol 1.5 g, ascorbic acid 4 g, casein 20 g, choline chloride 1 g, and H_2_O 800 mL. Adults were provided with fresh, tender *P. massoniana* branches. Larvae at each instar (L1–L5), pupae, and adults were collected, immediately frozen in liquid nitrogen, and stored at −80 °C for subsequent RNA extraction and qPCR analysis.

### 2.2. Genome-Wide Identification of MaltCPs

The *M. alternatus* genome assembly and annotation files were obtained from the insect genome database (http://v2.insect-genome.com/Genomeo, accessed on 20 January 2026). To identify cuticular protein (CP) genes, we adopted a multi-step homology- and structure-domain-based screening strategy. Profile Hidden Markov Models (pHMMs) for all known insect CP families, including CPR (PF00379), CPAP (PF01607), TWEEDLE (PF08917), CPF (PF11018), and CPCFC (PF17223), were retrieved from the Pfam database (http://xfam.org/, accessed on 26 January 2026) and used to search the *M. alternatus* proteome with HMMER 3.0 under a stringent E-value threshold of < 1 × 10^5^. To complement this, local BLASTp and tBLASTn searches were performed against the *M. alternatus* genome using previously reported CP sequences from other beetles as queries, with candidates retained at an E-value < 1 × 10^−5^ and sequence coverage > 50%. All candidates from both approaches were merged, subjected to redundancy removal, and validated via NCBI CDD and SMART databases to confirm the presence of canonical CP-associated conserved domains; sequences lacking recognizable CP domains were discarded. Additionally, keyword-based searches (“CP”, “CPR”, “CPAP”, “CPF”, and “Tweedle”) were conducted against transcriptome data to recover genes potentially absent from genome annotation due to assembly gaps or annotation errors, followed by cross-validation with genomic CDS sequences. Through these filtering steps, a final set of 119 non-redundant CP genes was obtained and classified into nine families based on conserved domain architectures and sequence similarity to known insect CPs. Among these, the CPAP family was further divided into CPAP1 (with one CBM_14 domain) and CPAP3 (with three CBM_14 domains) according to the number of chitin-binding domains (CBM_14) they contained. The remaining families were CPR-RR1, CPR-RR2, CPU, CPF, CPFL, TWEEDLE, and CPCFC.

### 2.3. Chromosomal Localization and Synteny Analysis

To determine the physical distribution of MaltCP genes on chromosomes, chromosomal location information (including chromosome number, start position, and end position) was extracted for all MaltCP genes based on the *M. alternatus* genome annotation file (GFF3 format). Chromosomal localization maps were generated using the “Gene Location Visualize” function of TBtools (version 1.098). Each MaltCP gene was plotted on its corresponding chromosome according to its actual physical position, with different CP families distinguished by distinct colors, to visualize the distribution density and regional preference of MaltCP genes across the nine chromosomes.

To investigate the evolutionary mechanisms underlying CP gene family expansion, MCScanX (version 1.0) was employed for genome-wide synteny analysis. First, BLASTp all-vs.-all self-alignment was performed on the full-length protein sequences of the *M. alternatus* genome, with an E-value threshold of 1 × 10^−5^ and a maximum of five hits retained. Based on the BLASTp results and the genome annotation files, MCScanX was run with default parameters to detect syntenic blocks within the genome. In the intraspecies synteny analysis, CP genes located within syntenic blocks were identified from the MCScanX output, including tandem duplication events (gene spacing < 200 kb) and segmental duplication events (duplications involving larger chromosomal regions), as listed in the .tandem file. For the interspecies synteny analysis, whole-genome protein sequences of *M. alternatus* were compared with those of *Tribolium castaneum* using BLASTp (E-value threshold 1 × 10^−5^). Combined with the genome annotation files of both species, MCScanX was run to detect inter-species syntenic blocks, enabling the assessment of lineage-specific expansion of CP gene families. In the synteny results, intraspecies syntenic blocks were used to identify duplication events and gene clusters, while interspecies syntenic blocks were used to compare the positional conservation of CP genes across genomes. All syntenic blocks and duplication events were visualized using the JCVI Python library (Python 3), generating syntenic circle plots (for intraspecies) and syntenic dot plots (for interspecies), with CP genes highlighted in distinct colors to facilitate intuitive identification of duplication patterns and inter-species syntenic relationships.

### 2.4. Conserved Domain and Phylogenetic Analysis

Conserved motif analysis of MaltCPs was performed using the MEME Suite (version 5.5.0) online tool. The analysis was conducted on the full-length amino acid sequences of all 119 identified MaltCP proteins, with the following parameters: maximum number of motifs set to 10, motif width ranging from 6 to 50 amino acids, and an E-value threshold of <0.05. The identified motifs were then annotated by comparison with previously reported conserved motifs in insect cuticular proteins using the motif databases available in MEME and by carrying out manual literature curation. The distribution patterns of these conserved motifs across different CP families were systematically examined to characterize family-specific sequence features and to assess the degree of motif conservation and divergence among families. For phylogenetic analysis, the protein sequences of MaltCPs were subjected to BLASTp searches against the NCBI non-redundant protein database to retrieve homologous sequences from other representative insect species. Multiple sequence alignments were performed using ClustalW (version 2.1) with default parameters. The best-fit amino acid substitution model was determined using the Model Selection module implemented in MEGA 11, based on the Bayesian Information Criterion (BIC). Phylogenetic trees were reconstructed using the Maximum Likelihood (ML) method in MEGA 11 with the selected best-fit model. Branch support was evaluated using 1000 bootstrap replicates. The resulting tree files were exported in Newick format and visualized using the interactive Tree of Life (iTOL) online platform (https://itol.embl.de/, accessed on 19 March 2026) for annotation and figure preparation. Bootstrap support values for major nodes are indicated on the phylogenetic trees.

### 2.5. Developmental-Stage-Specific Expression Profiles of MaltCP Genes

As a holometabolous insect, the life cycle of *M. alternatus* includes four developmental stages: egg, larva (1st–5th instar), pupa, and adult. To investigate the stage-specific expression profiles of MaltCP genes, RNA-seq data corresponding to the larval, pupal, and adult stages were retrieved and downloaded from the NCBI SRA database (https://www.ncbi.nlm.nih.gov/sra, accessed on 21 April 2026) under BioProject accession number PRJNA313481 (SRA run accessions: SRR3195361, SRR3195369, SRR3195370, SRR3195380, SRR3195381, SRR3195382, SRR3196146, SRR3196147, SRR3196148, SRR3196153, SRR3196167, and SRR3196177). Quantification of transcript abundances was performed using kallisto (version 0.48.0) with the following parameters: paired-end mode, sequence-based bias correction, and 100 bootstrap replicates (-b 100); all other parameters were set to default. The resulting transcript-level abundances were summarized at the gene level, and expression levels were calculated as transcripts per million (TPM) for each MaltCP gene. The TPM values were then used to generate heatmaps to visualize the expression patterns of MaltCP genes across different developmental stages. Additionally, the online tool ImageGP was employed to further analyze and visualize the expression profiles of MaltCP genes. Candidate genes showing high expression levels across stages were selected for further validation via quantitative real-time PCR (qRT-PCR).

### 2.6. RNA Extraction, cDNA Synthesis, and qRT-PCR

Total RNA was extracted from *M. alternatus* larvae, pupae, and adults using the HiPure Universal RNA Kit (Magen, Guangzhou, China). First-strand cDNA was synthesized using the All-in-One First-Strand Synthesis MasterMix (with dsDNase; Fujian Baimeng Medical Technology Co., Ltd., Fuzhou, China) according to the manufacturer’s instructions. Quantitative real-time PCR (qRT-PCR) was performed on a LightCycler480 II instrument. Each reaction was run in four biological replicates. The Maltβ-Actin gene was used as the reference gene for normalization, as it has been previously employed in qRT-PCR studies of *M. alternatus* [34]. Relative expression levels were calculated using the 2^−∆∆Ct^ method. The primers used for qRT-PCR are listed in Table 1.

### 2.7. Statistical Analysis

Data were organized and analyzed using Excel and SPSS 27, and graphs were generated using GraphPad Prism 10.1 software.

## 3. Results

### 3.1. Genome-Wide Identification of CP Genes in M. alternatus

A total of 119 CP genes were identified from the *M. alternatus* genome, belonging to nine families (CPR-RR1, CPR-RR2, Tweedle, CPAP1, CPAP3, CPF, CPFL, CPU, and CPCFC), with the CPR family being the most abundant (73 genes, 61.34%). To provide a broader evolutionary context, we compared the CP gene numbers identified in this study with those reported in representative species from six major insect orders (Table 2).

Among Coleoptera, the total CP gene counts ranged from 119 in *M. alternatus* to 173 in *L. decemlineata*, with *T. castaneum* having 144 genes and *Anoplophora glabripennis* (Motschulsky, 1854) having 166 genes; the CPR family (CPR-RR1 + CPR-RR2) varied from 84 in *T. castaneum* to 135 in *L. decemlineata*, and the CPR-RR2 subfamily consistently outnumbered CPR-RR1 in most Coleoptera species, particularly in *L. decemlineata* (85 vs. 50). Across other orders, CP gene numbers ranged from 63 in *Locusta migratoria* (Linnaeus, 1758) (Orthoptera) to 211 in *D. melanogaster* (Diptera), with *B. mori* (Lepidoptera) having 173 genes, *A. mellifera* (Hymenoptera) having 66 genes, and *Nilaparvata lugens* (Stål, 1854) (Hemiptera) having 133 genes; the relatively lower counts in *A. mellifera* and *L. migratoria* may reflect lineage-specific gene loss or differences in genome annotation completeness. Notably, the CP gene count in *M. alternatus* (119) falls within the range observed among other Coleoptera species, suggesting that its CP gene repertoire is comparable to that of other beetles. However, these comparisons should be interpreted with caution, as genome assembly quality and annotation completeness vary among the species compared.

### 3.2. Chromosomal Distribution and Synteny Analysis of MaltCP Genes

**Figure 1:**
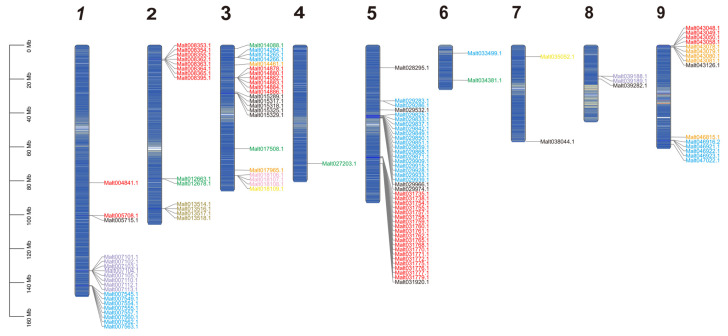
Chromosomal localization map of the cuticular protein gene family in *M. alternatus.* Colors indicate CP families: blue (CPR-RR1), red (CPR-RR2), green (CPAP1), orange (CPAP3), purple (Tweedle), gold (CPF), pink (CPFL), brown (CPCFC), and black (CPU).

To determine the physical distribution of MaltCP genes on chromosomes, a chromosomal localization map was generated using TBtools software (Figure 1). The distribution of MaltCP genes across the nine chromosomes of *M. alternatus* was uneven, with functionally related CP genes tending to cluster on specific chromosomes and forming distinct family-specific aggregation patterns. Chromosomes 1, 2, 3, 5, and 9 harbored multi-gene clusters, with chromosome 5 showing the most striking aggregation—a super-cluster comprising CPR-RR1, CPR-RR2, and CPU subtypes. Chromosome 3 exhibited the most diverse CP enrichment, with dense co-clustering of multiple CP subfamilies. By contrast, chromosomes 4, 6, and 7 contained only isolated CP genes or small clusters.

**Figure 2:**
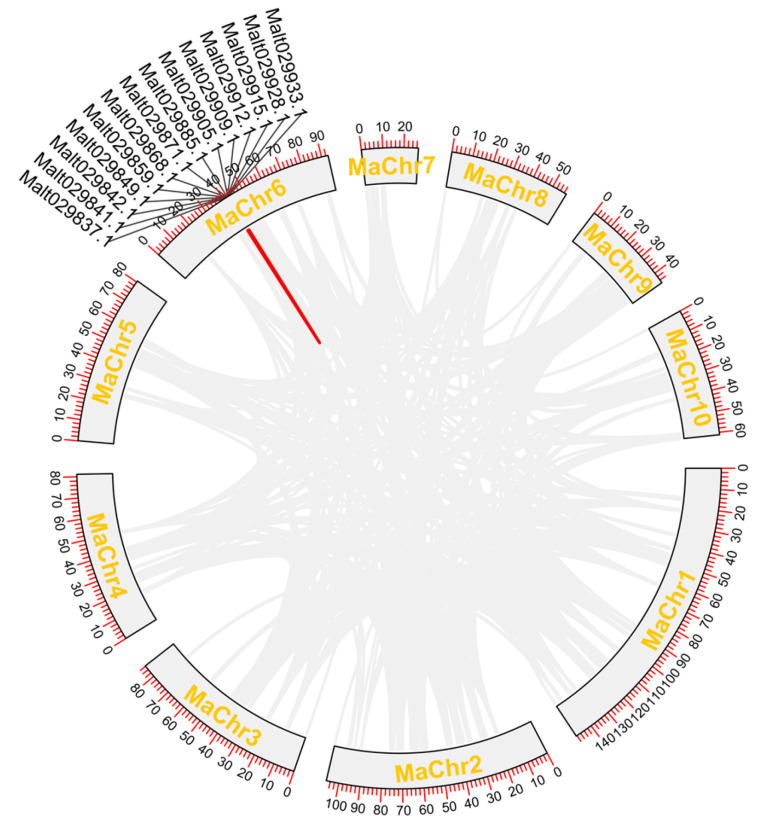
Intraspecies synteny analysis of CP genes in *M. alternatus*.

Intragenomic synteny analysis revealed seven tandem duplication events involving CP genes, all of which were localized on chromosome 6 (Figure 2). Specifically, the following seven gene pairs exhibited direct collinearity: Malt029837.1–Malt029933.1, Malt029841.1–Malt029928.1, Malt029842.1–Malt029915.1, Malt029849.1–Malt029912.1, Malt029859.1–Malt029909.1, Malt029868.1–Malt029905.1, and Malt029871.1–Malt029885.1. These seven pairs form distinct colinear blocks corresponding to tandem repeats, suggesting that localized duplications have occurred in this chromosomal region. The clustering of these tandemly duplicated CP genes on chromosome 6 indicates that local genome rearrangements have played a prominent role in shaping the organization of CP gene families in *M. alternatus*, potentially contributing to gene family expansion, functional redundancy, or neofunctionalization.

**Figure 3:**
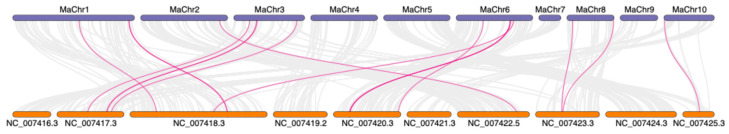
Interspecies synteny analysis of CP genes between *M. alternatus* and *T. castaneum*.

To assess the evolutionary conservation of CP gene families, interspecies synteny analysis was performed by comparing the genomes of *M. alternatus* and *T. castaneum* (Figure 3). The results revealed a total of 16 syntenic gene pairs distributed across six chromosomes of *M. alternatus*. Among these, MaChr6 harbored the highest number of syntenic pairs (five pairs), followed by MaChr3 (four pairs) and MaChr1 (three pairs), while MaChr8 contained two pairs, and MaChr2 and MaChr10 each contained one pair. The detailed pairing information is as follows: on MaChr3, NC_007417.3—Tcas012076.1 → Malt015289.1, NC_007417.3—Tcas011696.1 → Malt015317.1, NC_007417.3—Tcas012432.1 → Malt018106.1, and NC_007417.3—Tcas012451.1 → Malt014878.1; on MaChr1, NC_007418.3—Tcas000783.1 → Malt007545.1, NC_007418.3—Tcas000564.1 → Malt007554.1, and NC_007418.3—Tcas001103.1 → Malt004841.1; on MaChr6, NC_007418.3—Tcas000445.1 → Malt031920.1, NC_007420.3—Tcas004352.1 → Malt029283.1, NC_007420.3—Tcas004271.1 → Malt031735.1, NC_007420.3—Tcas004957.1 → Malt031738.1, and NC_007420.3—Tcas004969.1 → Malt031768.1; on MaChr2, NC_007422.5—Tcas008361.1 → Malt013514.1; on MaChr8, NC_007423.3—Tcas009184.1 → Malt035052.1, and NC_007423.3—Tcas010482.1 → Malt038044.1; and on MaChr10, NC_007425.3—Tcas010684.1 → Malt043058.1. The widespread distribution of syntenic events across multiple chromosomes indicates significant genomic structural conservation between the two species. Notably, MaChr6, MaChr3, and MaChr1 carried more syntenic pairs, suggesting that these chromosomal regions are highly conserved during evolution and may contain cross-species conserved duplication blocks or functional gene segments. These findings provide genomic evidence for the evolutionary conservation and lineage-specific expansion of CP gene families.

### 3.3. CPR Family Genes

This study identified 73 CPR family genes containing the conserved R&R motif, constituting 61.34% of the total CP genes in *M. alternatus*. Based on the transcriptome data of *M. alternatus* and using BLAST homology search, the CPR family was found to include 34 CPR-RR1 subfamily members and 39 CPR-RR2 subfamily members.

**Figure 4:**
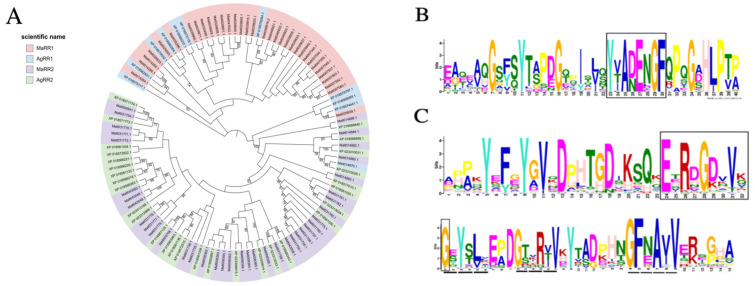
Phylogenetic and conserved sequence analysis of CPR family proteins in *M. alternatus,* including *Monochamus alternatus* (MaRR1; MaRR1) and *Anoplophora glabripennis* (AgRR1; AgRR2). The phylogenetic tree was reconstructed using the Maximum Likelihood (ML) method implemented in MEGA 11 with the best-fit model selected based on BIC. Bootstrap support values (≥70%) are shown at the branch nodes (**A**). Conserved domain analysis of CPR-RR1 proteins (**B**). Conserved domain analysis of CPR-RR2 proteins (**C**). Conserved motifs were identified using MEME Suite (version 5.5.0) with the following parameters: maximum number of motifs set to 10, motif width ranging from 6 to 50 amino acids, and an E-value threshold of <0.05. Each motif is represented by a colored box, and the order of boxes corresponds to the motif positions along the protein sequences.

A phylogenetic tree of the *M. alternatus* CPR family was constructed using the Maximum Likelihood (ML) method. Conserved domain analysis of CPR-RR1 and CPR-RR2 family proteins was also performed (Figure 4).

CPR-RR1 family proteins were found to possess eight conserved amino acid recognition sites (YTADENGF) (Figure 4B), whereas CPR-RR2 family proteins contained two conserved recognition sequences: EERDGDVVKG and three G-X (3)-VV repeat motifs (Figure 4C).

To further investigate the evolutionary relationships of insect CPR-RR1 and CPR-RR2 proteins, a phylogenetic analysis was conducted using protein sequences from *A. glabripennis* and *M. alternatus* and a phylogenetic tree of CPR family proteins from different insects was constructed (Figure 4A). The results showed that the *M. alternatus* CPR-RR1 family genes formed a single clade, while the CPR-RR2 family protein genes formed another distinct clade.

### 3.4. CPAP1 and CPAP3 Family Genes

**Figure 5:**
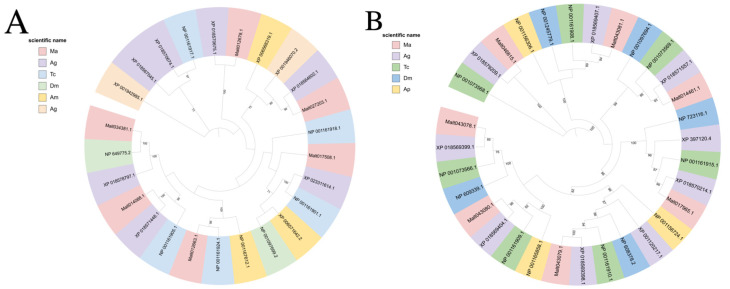
Evolutionary relationships of CPAP1 and CPAP3 family proteins from different insects. (**A**) Evolutionary relationships of CPAP1 family proteins from different insects, including *Monochamus alternatus* (Ma), *Anoplophora glabripennis* (Ag), *Tribolium castaneum* (Tc), *Drosophila melanogaster* (Dm), *Apis mellifera* (Am), and *Acyrthosiphon pisum* (Ap). (**B**) Evolutionary relationships of CPAP3 family proteins from different insects, including *Monochamus alternatus* (Ma), *Anoplophora glabripennis* (Ag), *Drosophila melanogaster* (Dm), *Tribolium castaneum* (Tc), and *Acyrthosiphon pisum* (Ap). The phylogenetic tree was reconstructed using the Maximum Likelihood (ML) method implemented in MEGA 11 with the best-fit model selected based on BIC. Bootstrap support values (≥70%) are shown at the branch nodes.

CPAP1 and CPAP3 contain a peritrophin-A motif with six regularly spaced cysteine residues, and one and three ChtBD2 domains, respectively. A total of six CPAP1 and seven CPAP3 genes were identified in the *M. alternatus* genome. Phylogenetic analysis showed that CPAP1 and CPAP3 family proteins from different insects formed distinct clades. *Monochamus alternatus* showed a closer phylogenetic relationship with *A. glabripennis* and *T. castaneum*. Except for Malt034381.1 from the CPAP1 family, all CPAP genes from *M. alternatus* formed pairs with their orthologs from other Coleoptera insects in the evolutionary tree (Figure 5).

### 3.5. Tweedle Family Genes

A total of 10 Tweedle genes were identified in the *M. alternatus* genome. A phylogenetic tree of the Tweedle family proteins was constructed based on the amino acid sequences of *M. alternatus*, *T. castaneum*, and four other insect species. The results showed that most Tweedle family genes of *M. alternatus* clustered with those of *T. castaneum*, indicating a close homology. Malt007102.1 and Malt007103.1 showed over 90% amino acid sequence identity with A. gambiae and A. aegypti. In contrast, Malt039189.1 showed over 70% amino acid sequence identity with the other five species in terms of evolutionary relationship (Figure S1A).

Furthermore, amino acid sequence analysis revealed that the Tweedle family proteins of *M. alternatus* possess two conserved amino acid regions, and each Tweedle family member contains an internal repeat structure (Figure S1B).

### 3.6. CPF and CPFL Family Genes

A total of two CPF and three CPFL genes were identified in the *M. alternatus* genome. Phylogenetic trees were constructed separately based on the protein sequences of CPF and CPFL from different insects. The results showed that the proteins of these two families clustered to form two parallel branches (Figure S2A). Sequence analysis revealed that the CPF proteins of Coleoptera insects *M. alternatus*, *T. castaneum*, and *Sitophilus oryzae* (Linnaeus, 1763) each contain conserved 44-amino acid and C-terminal sequences (Figure S2B), while the CPFL family proteins only possess a C-terminal conserved sequence with high homology to CPF proteins (Figure S2C).

### 3.7. CPCFC Family Genes

In this study, a total of four CPCFC genes were annotated in the *M. alternatus* genome. To clarify the phylogenetic relationships of CPCFC proteins, phylogenetic analysis was performed using CPCFC protein sequences from five Coleoptera and four Diptera insect species. The results showed that CPCFC proteins from Coleoptera insects clustered together, and those from Diptera insects formed another cluster (Figure S3A). Amino acid sequence analysis of CPCFC proteins from *M. alternatus*, *T. castaneum*, and other Coleoptera insects revealed that this family of proteins shares a common conserved domain, namely two repeated C–X (5)–C motifs (Figure S3B).

### 3.8. CPU Family Genes

A total of 14 CPU family genes were identified in the *M. alternatus* genome, which is the same number as in *Xylotrechus colonus* (Fabricius, 1775) and one more than in *A. glabripennis*. The conserved motifs of CPU family cuticular protein amino acid sequences from *M. alternatus* and *A. glabripennis* were analyzed using the online tool MEME. The analysis results showed that the conserved motif for the CPU family genes in *M. alternatus* is G-X-Y-X (3)-D-X (2)-G-X (6)-Y (Figure S4).

### 3.9. Expression Profile Analysis and qPCR Validation of Cuticular Protein Genes in M. alternatus

**Figure 6:**
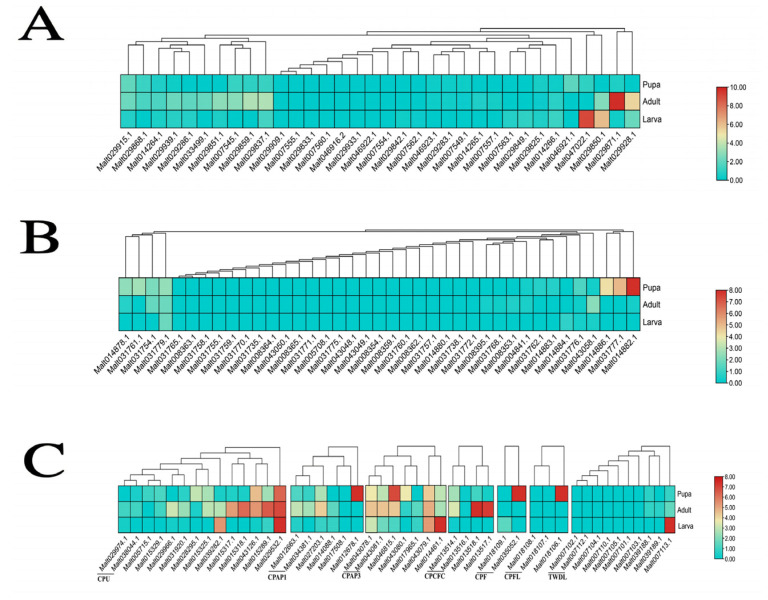
Heatmap of expression levels of MaltCP genes from different families in the pine sawyer beetle (*M. alternatus*) at various developmental stages. Expression profiles of CPR-RR1 family genes in *M. alternatus* under different states, showing higher expression of Malt029850.1, Malt047022.1, Malt029928.1, and Malt029871. (**A**). Expression profiles of CPR-RR2 family genes in *M. alternatus* under different states, showing higher expression of Malt014886.1, Malt031777.1, and Malt014882.1 (**B**). Expression profiles of CPU, CPAP1, CPAP3, CPCFC, CPF, CPFL, and TWEEDLE family genes in *M. alternatus* under different states, showing stage-preferred expression patterns across larval, pupal, and adult stages.  The color scale represents expression levels (**C**).

Based on the transcriptomic BioProject data of *M. alternatus*, the expression levels of the identified MaltCP genes at different developmental stages were analyzed using ImageGP. The results (Figure 6) showed that within the CPR-RR1 family genes, Malt029850.1, Malt047022.1, Malt029928.1, and Malt029871.1 exhibited higher expression levels (Figure 6A). In the CPR-RR2 family genes, Malt014886.1, Malt031777.1, and Malt014882.1 showed higher expression (Figure 6B). In the CPU family, Malt029532.1 had high expression across larval, pupal, and adult stages; Malt043126.1 showed high expression during pupal and adult stages; Malt015317.1, Malt015318.1, and Malt015289.1 had high expression in the adult stage; and Malt039282.1 was highly expressed during the larval stage. In the CPAP1 family, Malt012678.1 was highly expressed during the pupal stage, and Malt027203.1 was highly expressed in the adult stage. The seven selected CPAP3 family genes all showed relatively high expression, but their expression levels varied significantly across different developmental stages. In the CPCFC family, Malt013518.1 and Malt013517.1 exhibited higher expression in the adult stage. The CPF family gene Malt035052.1 and the CPFL family gene Malt018106.1 showed higher expression during the pupal stage. The TWEEDLE family gene Malt007113.1 was highly expressed in the larval stage (Figure 6C). In summary, the expression abundance of cuticular protein genes from different families in *M. alternatus* varied considerably across developmental stages.

**Figure 7:**
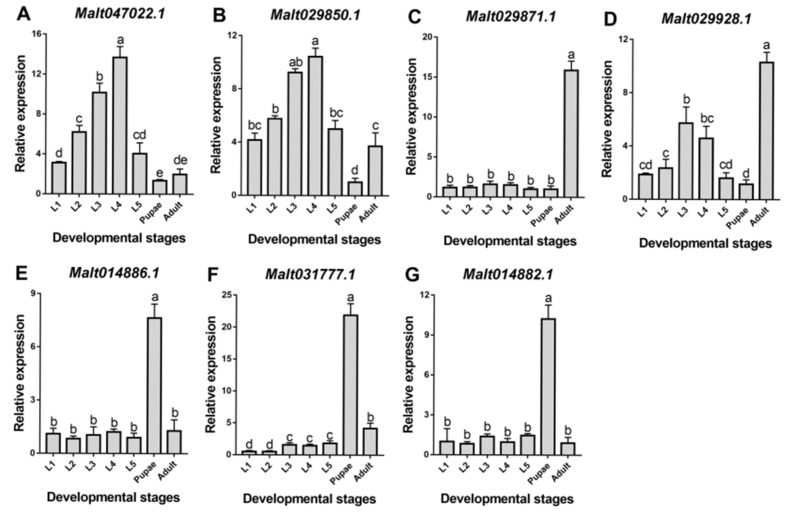
qPCR validation of expression levels of CPR family genes in the pine sawyer beetle (*M. alternatus*) across the 1st- to 5th-instar larva (L1–L5), pupa, and adult stages. Expression profiles of CPR-RR1 family genes (highly expressed) (**A**–**D**) and CPR-RR2 family genes (highly expressed) (**E**–**G**) are shown. Different lowercase letters above bars indicate significant differences among developmental stages (*p* < 0.05), whereas the same letter indicates no significant difference.

To prioritize candidates for qPCR validation, we selected MaltCP genes that exhibited both high transcript abundance and clear stage-specific expression patterns based on the RNA-seq data (Figure 6A,B). Specifically, four CPR-RR1 genes (Malt029850.1, Malt047022.1, Malt029928.1, and Malt029871.1) and three CPR-RR2 genes (Malt014886.1, Malt031777.1, and Malt014882.1) were chosen, as they showed the most pronounced differential expression across larval, pupal, and adult stages. These genes represent promising candidates involved in cuticle formation and development; their validation by qPCR serves both to corroborate the transcriptomic results and to establish a foundation for future functional studies using RNA interference. The qPCR results revealed that the seven selected CPR family genes exhibited distinct expression patterns across different developmental stages, which could be classified into four types based on their expression trends. The first type (A: Malt047022.1; B: Malt029850.1) exhibited gradually increasing expression from L1 to L4, peaking at L4, followed by a sharp decline to the lowest levels in pupae and remaining relatively low in adults, suggesting a role for these genes in larval development and molting. The second type (C: Malt029871.1) showed extremely low expression across all larval instars and the pupal stage, with no significant differences among these stages, but its expression increased sharply in adults, significantly exceeding all other stages, suggesting a potential role in adult-specific physiological processes. The third type (D: Malt029928.1) showed a sub-peak at L3, reached the lowest level in pupae, and then increased sharply to the highest level in adults, displaying dual expression characteristics in both larval and adult stages. The fourth type (E: Malt014886.1; F: Malt031777.1; G: Malt014882.1) exhibited pupa-specific high expression, with low levels across all larval instars and the adult stage but significantly elevated expression in pupae, reaching the highest peak across all stages, among which gene F (Malt031777.1) showed a relative expression level exceeding 20 in pupae, suggesting their involvement in pupal cuticle remodeling and metamorphosis. Overall, the qPCR validation results were largely consistent with the differential expression patterns observed in the transcriptomic analysis (Figure 7).

## 4. Discussion

As crucial structural components of the insect cuticle, CPs are not only numerous in type and quantity but also play key roles in insect growth, development, and environmental adaptation, making them ideal targets for developing novel pest control agents [3,42]. Therefore, gaining an in-depth understanding of the cuticular protein genes in *M. alternatus* and their expression patterns across different developmental stages can provide new insights for developing control strategies targeting the cuticle of *M. alternatus*.

Insect cuticular proteins constitute a large family, with the CPR family being the most widespread and numerous, accounting for approximately 70% of all ICPs. Its members have been found in insects of Diptera, Lepidoptera, Coleoptera, Hymenoptera, Hemiptera, and Orthoptera [43]. The distribution of MaltCP genes across the nine chromosomes of *M. alternatus* is uneven, with chromosomes 1, 2, 3, 5, and 9 harboring more MaltCP genes, while the remaining chromosomes irregularly contain one to four genes. Based on the *M. alternatus* genome, a total of 119 CP genes were identified, belonging to nine families: CPR-RR1, CPR-RR2, Tweedle, CPAP1, CPAP3, CPF, CPFL, CPU, and CPCFC. The CPR family comprises 73 genes, the highest number, accounting for 61.34% of the total CP genes in *M. alternatus*. The predominance of the CPR family is consistent with findings in other Coleoptera species, such as *T. castaneum* and *A. glabripennis* [15,44], suggesting a conserved role of this family in cuticle formation across the order. Intraspecies synteny analysis identified seven tandem duplication events, all localized on chromosome 6, suggesting that tandem duplication is a major driving force for CP gene family expansion in this species. Interspecies synteny analysis between *M. alternatus* and *T. castaneum* revealed 16 conserved syntenic gene pairs distributed across six chromosomes, indicating significant genomic structural conservation between the two species and providing evidence for lineage-specific expansion of certain CP subfamilies. Collectively, these findings lay a solid foundation for future functional studies of CP genes in *M. alternatus* and offer valuable insights for developing environmentally friendly pest management strategies targeting the insect cuticle.

The observed variations in CP family sizes among Coleoptera species warrant careful interpretation. While we found that the CPR-RR1 and CPFL family gene numbers in *M. alternatus* are comparable to those in *T. castaneum*, and the CP-RR2 family gene number is higher than in *C. mutillarius* but lower than in *T. castaneum*, several factors must be considered before attributing these differences to ecological adaptation. First, the genome assembly quality and annotation completeness vary considerably among species—for instance, the *A. glabripennis* genome [44] and *L. decemlineata* genome [21] were assembled using different sequencing technologies and annotation pipelines, which may influence gene prediction sensitivity. Second, differences in CP gene family classification criteria across studies (e.g., the use of different E-value thresholds or domain models) can directly affect gene counts. Third, the absence or presence of specific CP subfamilies may also reflect lineage-specific gene loss or gain events rather than adaptive responses to environmental conditions. Therefore, while the observed differences in CP gene numbers across Coleoptera species are intriguing, future studies incorporating standardized annotation pipelines and functional assays are needed to determine whether these variations have adaptive significance. We have acknowledged these limitations in the present study to avoid overinterpretation of the observed differences [45,46,47].

CPAP1 and CPAP3 have a peritrophin-A motif with six distinctly spaced cysteine residues, and one and three ChtBD2 domains, respectively [17]. The CPF proteins of coleopteran insects *M. alternatus*, *T. castaneum*, and *S. oryzae* contain conserved 44-amino acid and C-terminal sequences, respectively, while the CPFL family proteins only possess C-terminal conserved sequences with high homology to CPF proteins [27]. The Tweedle family proteins in *M. alternatus* possess two conserved amino acid regions, and each Tweedle family member contains an internal repeat structure [48,49,50]. Amino acid sequence analysis of the CPCFC family in coleopteran insects like *M. alternatus* and *T. castaneum* revealed that proteins in this family share a common conserved domain, namely two repeated C-X (5)-C motifs [12,14]. A total of 14 CPU family genes were identified in the *M. alternatus* genome, a number equal to that in *C. mutillarius* and one more than in *A. glabripennis* [51,52,53]. Using the online tool MEME, conserved motif analysis was performed on the amino acid sequences of CPU family cuticular proteins from *M. alternatus* and *A. glabripennis*. The results showed that the conserved motif for the CPU family genes in *M. alternatus* is G-X-Y-X(3)-D-X(2)-G-X(6)-Y.

The expression abundance of cuticular protein genes from different families in *M. alternatus* varied considerably across developmental stages [44,54], and these stage-specific patterns provide insights into the distinct roles of CP families during cuticle formation and metamorphosis. To validate the transcriptomic data, seven MaltCP genes from the CPR family were selected for qPCR analysis. The results indicated that within the CPR-RR1 family, the expression levels of Malt029850.1 and Malt047022.1 were higher in larvae than in pupae and adults, while the expression levels of Malt029928.1 and Malt029871.1 were higher in adults than in pupae and larvae. In the CPR-RR2 family genes, the expression levels of Malt014886.1, Malt031777.1, and Malt014882.1 were significantly higher during the pupal stage compared to the other two stages. The qPCR validation results were largely consistent with the differential expression patterns observed in the transcriptomic analysis [9,26].

The stage-specific expression patterns observed in this study can be interpreted in the context of cuticle formation and sclerotization during metamorphosis [21]. The qPCR results revealed that the seven CPR family genes exhibited distinct expression patterns across different developmental stages, which could be classified into four types: A (Malt047022.1) and B (Malt029850.1) peaked at L4 and declined to the lowest levels in pupae, suggesting their involvement in larval development and molting; C (Malt029871.1) showed high expression exclusively in adults, with extremely low levels in larvae and pupae, suggesting a potential role in adult-specific physiological processes; D (Malt029928.1) displayed two expression peaks at the L3 and adult stages, exhibiting dual characteristics in both larval and adult stages; E (Malt014886.1), F (Malt031777.1), and G (Malt014882.1) exhibited pupa-specific high expression, with gene F showing a relative expression level exceeding 20 in pupae, suggesting their involvement in pupal cuticle remodeling and metamorphosis [55,56].

The life activities of *M. alternatus* at different instars are closely related to their physiological states [21]. Female adults prefer to oviposit on weakened pine trees, likely due to lower host resistance favoring offspring survival. Empty grooves are frequently observed among the feeding scars, possibly because not every groove is suitable for larval development or because the beetles encounter unfavorable environmental factors during groove formation. After hatching, first- and second-instar larvae feed beneath the bark, third-instar larvae bore into the phloem, fourth-instar larvae enter the xylem, and fifth-instar larvae pupate and emerge as adults [55]. The instar-specific expression patterns revealed by our qPCR results are highly consistent with the biological characteristics of *M. alternatus*. During the larval stages (L1–L5), genes A and B peaked at L4, which is the critical stage when larvae transition from the phloem to the xylem, where they encounter high concentrations of terpenoids from the host plant. The high expression of CP genes at this stage may be involved in cuticle reinforcement to adapt to the xylem environment [57]. *M. alternatus* shows the strongest resistance to α-pinene at the fourth instar, which may be associated with exposure to higher terpenoid levels upon entering the xylem. The elevated expression of genes A and B at L4 may reflect the critical role of cuticular proteins in larval adaptation to the xylem environment [58]. Adults exhibit extremely high sensitivity to α-pinene, possibly because they no longer reside in terpenoid-rich pine tissues after eclosion and do not need to cope with high terpenoid concentrations as larvae do. Meanwhile, adults possess rapid crawling and flight capabilities, enabling them to escape from unfavorable terpenoid environments, and their life mission has shifted from growth and development to reproduction. The high expression of gene C in adults is consistent with this life-stage transition and may be involved in adult eclosion and adult-specific physiological functions [40,59]. High terpenoid concentrations indicate high host resistance, which is unfavorable for offspring development—this may be the evolutionary reason for the high sensitivity of adults to α-pinene. During the pupal stage, the specific high expression of the three CPR-RR2 genes (E, F, and G) is closely associated with pupal cuticle remodeling and sclerotization, as the pupal stage represents the metamorphic transition from larva to adult, and the role of CPR-RR2 proteins in rigid cuticle formation has been widely established. Taken together, these stage-specific CP gene expression patterns are closely linked to the ecological adaptation strategies and physiological demands of *M. alternatus* at different developmental stages, providing an important foundation for understanding the developmental biology of this species and for developing cuticle-based pest management strategies [25].

We acknowledge that although our expression analysis covered first- to fifth-instar larvae (L1–L5), pupae, and adults, the egg stage was not included, and finer temporal sampling within each instar was not performed. Future studies with more comprehensive temporal sampling would be valuable to fully characterize the expression dynamics of CP genes throughout the entire life cycle. While the identified CP genes represent promising targets for pest management, it is important to acknowledge that their functional validation has not yet been performed. RNAi-based knockdown experiments, gene editing, or phenotypic analyses are required to confirm the biological significance of these genes in cuticle development, molting, and survival [60,61,62].

## 5. Conclusions

In summary, this study identified and characterized the MaltCP genes of *M. alternatus* through genome-wide analysis, examined their conserved domains, phylogenetic relationships, and syntenic organization, and compared their transcriptional levels across different developmental stages. The synteny and duplication analyses revealed that tandem duplication and lineage-specific expansion have played important roles in the evolution of the CP gene family in this species. This provides a reference for elucidating the physiological functions of MaltCP in *M. alternatus* and offers a new perspective for environmentally friendly pest control strategies. However, the CP genes identified in this study should be considered promising candidate targets for future functional validation rather than validated control agents. Future work will involve using RNA interference methods to study the effects of pesticide inhibition on *M. alternatus* when MaltCP expression is silenced, which will be essential to establish the causal relationship between CP gene function and insect survival.

## Figures and Tables

**Table 1:** Primer sequences used in this research.

Gene ID in InsectBase	Sequence (5′ → 3′)		Purpose
	Forward	Reverse	
Malt047022.1	AATCCCCATCATCAAGCAGG	AGGTGTAAGAGAAGGCACCG	qPCR
Malt029850.1	AAGCAGGACTCAGAAGTGAA	TGGAGAGGTGTAAGAGAAGG	qPCR
Malt029871.1	GCGAGGGAACAAGGGCGTTT	ATTGGTGGTGCTGTGGGCAA	qPCR
Malt029928.1	GCGAGGGAACAAGGGCGTTT	ATTGGTGGTGCTGTGGGCAA	qPCR
Malt014886.1	AATTTGCTTATGCTGCTCCC	ACATCTCCATTCCTGGTTTC	qPCR
Malt031777.1	ATTAGGTCTAGGAGGAGGGT	TTCAGTTTGCTGCTTTTGGT	qPCR
Malt014882.1	GACCATATTGACTGCTTTCG	CTTGACTCTTGCTGTCTCCC	qPCR
Maltβ-Actin	CGCCCCATCCACCATGAAGA	AGAGGGAGGCGAGGATGGAT	qPCR

The gene IDs listed in this table correspond to the identifiers annotated in the InsectBase database (http://v2.insect-genome.com/Genomeo). The protein and CDS sequences of the MaltCP genes can be retrieved from the InsectBase genome browser using these IDs.

**Table 2:** Comparison of cuticle protein gene numbers across insect orders.

Order	Species	CPR-RR1	CPR-RR2	CPAP1	CPAP3	CPCFC	CPF	CPFL	TWEEDLE	CPU	Total Number	Reference
Coleoptera	*Monochamus alternatus*	34	39	6	7	4	2	3	10	14	119	The present study
	*Anoplophora glabripennis*	59	66	7	7	4	3	0	7	13	166	[35]
	*Leptinotarsa decemlineata*	50	85	9	7	2	5	4	9	2	173	[36]
	*Tribolium castaneum*	34	55	15	7	2	5	3	2	21	144	[5]
Diptera	*Aedes aegypti*	66	150	14	9	1	3	12	6	28	289	[37]
	*Anopheles gambiae*	43	103	13	10	1	4	7	12	21	214	[38]
	*Drosophila melanogaster*	61	42	29	10	1	5	0	29	34	211	[39]
Hymenoptera	*Apis mellifera*	13	15	15	7	0	4	0	2	10	66	[5]
	*Nasonia vitripennis*	19	32	16	6	0	5	0	2	18	98	[5]
Lepidoptera	*Bombyx mori*	47	78	13	6	1	1	4	4	19	173	[7]
	*Danaus plexippus*	47	57	16	10	1	1	4	5	18	159	[5]
Hemiptera	*Vilaparvata lugens*	36	57	17	8	0	3	9	3	0	133	[40]
Orthoptera	*Locusta migratoria*	25	18	2	7	0	9	0	2	0	63	[41]

## Data Availability

The datasets used and/or analyzed in the current study are available from the corresponding author on reasonable request.

## Supplementary Materials

The following supporting information can be downloaded at: https://www.mdpi.com/article/10.3390/biology15141174/s1, Table S1: BioProject and SRA run accession numbers for the RNA-seq datasets used in this study; Figure S1. Evolutionary relationships and conserved sequence analysis of Tweedle family proteins from different insects. Analysis of the evolutionary relationships of insect Tweedle family proteins, including *Monochamus alternatus*, *Tribolium castaneum*, *Apis mellifera*, *Drosophila melanogaster*, *Anopheles gambiae*, and *Aedes aegypti* (A). The phylogenetic tree was reconstructed using the Maximum Likelihood (ML) method implemented in MEGA 11 with the best-fit model selected based on BIC. Bootstrap support values (≥70%) are shown at the branch nodes. Conserved domain analysis of Tweedle proteins in *Monochamus alternatus* (B). Conserved motifs were identified using MEME Suite (version 5.5.0) with the following parameters: maximum number of motifs set to 10, motif width ranging from 6 to 50 amino acids, and an E-value threshold of <0.05. Each motif is represented by a colored box, and the order of boxes corresponds to the motif positions along the protein sequences; Figure S2. Evolutionary relationship and conserved sequence analysis of CPF and CPFL family proteins from different insects. Analysis of the evolutionary relationships of insect CPF and CPFL family proteins, including *Monochamus alternatus* (Ma), *Tribolium castaneum* (Tc), and *Sitophilus oryzae* (So) (A). Conserved domain analysis of CPF proteins in *Monochamus alternatus***,** *Tribolium castaneum*, and *Sitophilus oryzae* (B). Conserved domain analysis of CPFL proteins in *Monochamus alternatus* and *Sitophilus oryzae* (C). Conserved motifs were identified using MEME Suite (version 5.5.0) with the following parameters: maximum number of motifs set to 10, motif width ranging from 6 to 50 amino acids, and an E-value threshold of <0.05. Each motif is represented by a colored box, and the order of boxes corresponds to the motif positions along the protein sequences; Figure S3. Evolutionary relationships and conserved sequence analysis of CPCFC family proteins from different insects. Analysis of the evolutionary relationships of insect CPCFC family proteins, including *Monochamus alternatus* (Ma), *Anoplophora glabripennis* (Ag), *Tribolium castaneum* (Tc), *Sitophilus oryzae* (So), *Dendroctonus ponderosae* (Dp), *Drosophila melanogaster* (Dm), *Anopheles gambiae* (Aga), *Anopheles funestus* (Af), and *Aedes aegypti* (Aa) (A). Conserved domain analysis of Tweedle proteins in *Monochamus alternatus* (B); Figure S4. Conserved sequence analysis of CPU family proteins in different insects. Conserved motifs were identified using MEME Suite (version 5.5.0) with the following parameters: maximum number of motifs set to 10, motif width ranging from 6 to 50 amino acids, and an E-value threshold of <0.05. Each motif is represented by a colored box, and the order of boxes corresponds to the motif positions along the protein sequences.
