# Supplementary material for: Identification and Expression Pattern Analysis of Cuticular Protein Gene Family in Monochamus alternatus (Coleoptera: Cerambycidae)
Source: Biology (Basel). 2026 Jul 16;15(14):1174. doi: 10.3390/biology15141174 (PMC13405353; doi:10.3390/biology15141174)
Supplement: Supplementary file 1 [file biology-15-01174-s001.zip › biology-4388189-supplementary.pdf]

Table S1. BioProject and SRA run accession numbers for the RNA-seq datasets used in this study.

**BioProject**	**SRA Run Accession (SRR)**
PRJNA313481	SRR3195361
PRJNA313481	SRR3195369
PRJNA313481	SRR3195370
PRJNA313481	SRR3195380
PRJNA313481	SRR3195381
PRJNA313481	SRR3195382

PRJNA313481	SRR3196146
PRJNA313481	SRR3196147
PRJNA313481	SRR3196148
PRJNA313481	SRR3196153
PRJNA313481	SRR3196167
PRJNA313481	SRR3196177

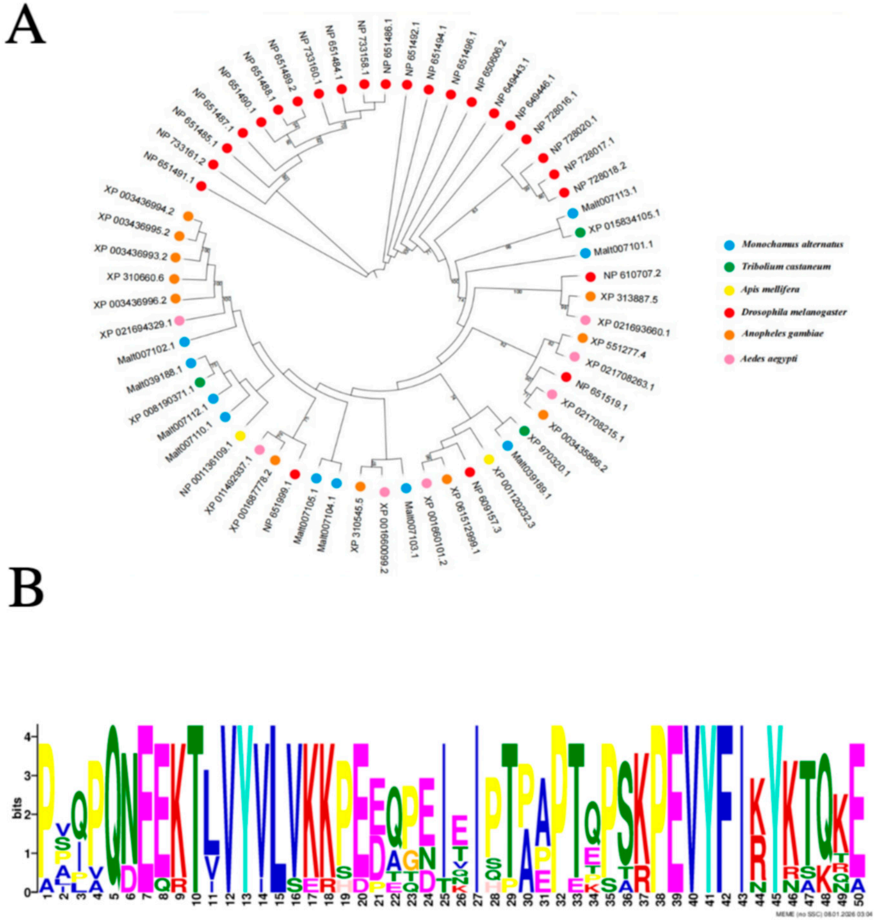

Figure S1. Evolutionary relationships and conserved sequence analysis of Tweedle family proteins from different insects. Analysis of the evolutionary relationships of insect Tweedle family proteins, including *Monochamus alternatus*, *Tribolium castaneum*, *Apis mellifera*, *Drosophila melanogaster*, *Anopheles gambiae*, and *Aedes aegypti* (A). The phylogenetic tree was reconstructed using the Maximum Likelihood (ML) method implemented in MEGA 11 with the best-fit model selected based on BIC. Bootstrap support values (≥ 70%)

are shown at the branch nodes. Conserved domain analysis of Tweedle proteins in *Monochamus alternatus* (B). Conserved motifs were identified using MEME Suite (version 5.5.0) with the following parameters: maximum number of motifs set to 10, motif width ranging from 6 to 50 amino acids, and an E-value threshold of < 0.05. Each motif is represented by a colored box, and the order of boxes corresponds to the motif positions along the protein sequences.

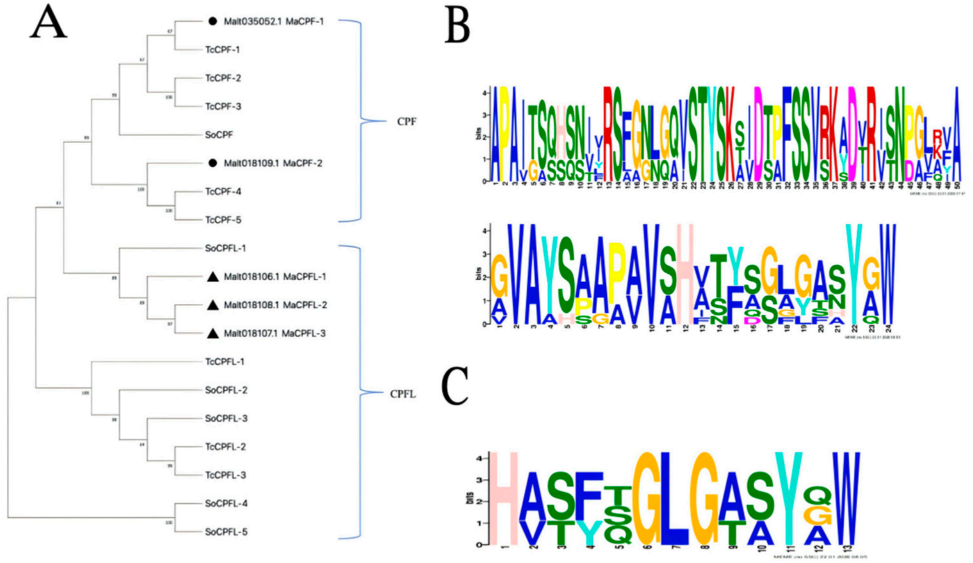

Figure S2. Evolutionary relationship and conserved sequence analysis of CPF and CPFL family proteins from different insects. Analysis of the evolutionary relationships of insect CPF and CPFL family proteins, including *Monochamus alternatus* (Ma), *Tribolium castaneum* (Tc), and *Sitophilus oryzae* (So) (A). Conserved domain analysis of CPF proteins in *Monochamus alternatus*, *Tribolium castaneum*, and *Sitophilus oryzae* (B). Conserved domain analysis of CPFL proteins in *Monochamus alternatus* and *Sitophilus oryzae* (C). Conserved motifs were identified using MEME Suite (version 5.5.0) with the following parameters: maximum number of motifs set to 10, motif width ranging from 6 to 50 amino acids, and an E-value threshold of < 0.05. Each motif is represented by a colored box, and the order of boxes corresponds to the motif positions along the protein sequences.

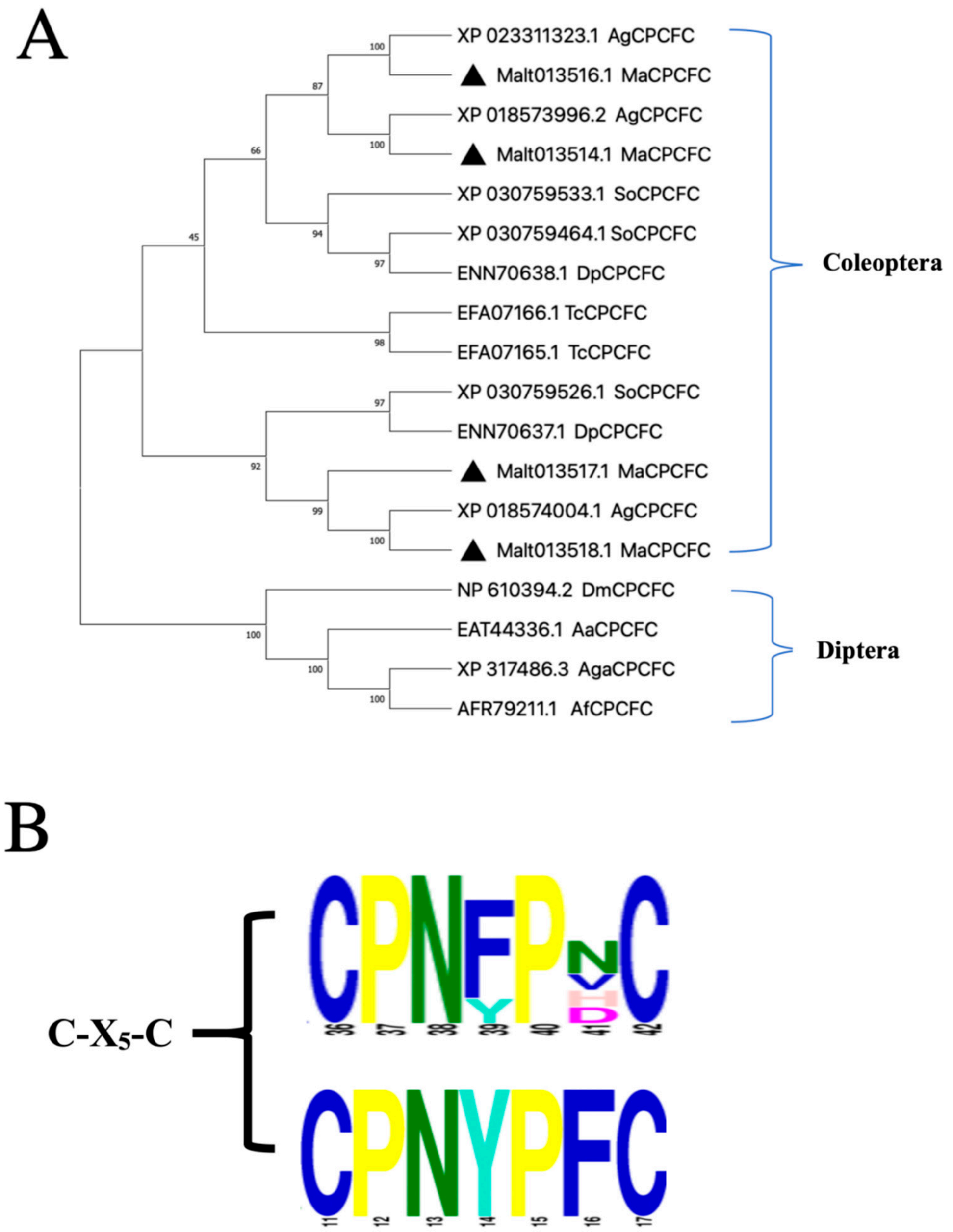
A
XP 023311323.1 AgCPCFC
Malt013516.1 MaCPCFC
XP 018573996.2 AgCPCFC
Malt013514.1 MaCPCFC
XP 030759533.1 SoCPCFC
XP 030759464.1 SoCPCFC
ENN70638.1 DpCPCFC
EFA07166.1 TcCPCFC
EFA07165.1 TcCPCFC
XP 030759526.1 SoCPCFC
ENN70637.1 DpCPCFC
Malt013517.1 MaCPCFC
XP 018574004.1 AgCPCFC
Malt013518.1 MaCPCFC
NP 610394.2 DmCPCFC
EAT44336.1 AaCPCFC
XP 317486.3 AgaCPCFC
AFR79211.1 AfCPCFC
Coleoptera
Diptera
B
C-X5-C

Figure S3. Evolutionary relationships and conserved sequence analysis of CPCFC family proteins from different insects. Analysis of the evolutionary relationships of insect CPCFC family proteins, including *Monochamus alternatus* (Ma), *Anoplophora glabripennis* (Ag), *Tribolium castaneum* (Tc), *Sitophilus oryzae* (So), *Dendroctonus ponderosae* (Dp), *Drosophila melanogaster* (Dm), *Anopheles gambiae* (Aga), *Anopheles funestus* (Af), and *Aedes aegypti* (Aa) (A). Conserved domain analysis of Tweedle proteins in *Monochamus alternatus* (B).

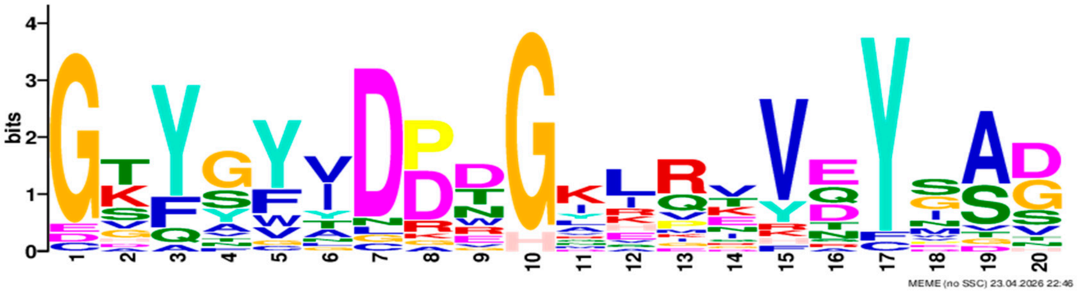

Figure S4. Conserved sequence analysis of CPU family proteins in different insects. Conserved motifs were identified using MEME Suite (version 5.5.0) with the following parameters: maximum number of motifs set to 10, motif width ranging from 6 to 50 amino acids, and an E-value threshold of < 0.05. Each motif is represented by a colored box, and the order of boxes corresponds to the motif positions along the protein sequences.
